# Nutritional, Texture, and Sensory Properties of composite biscuits produced from breadfruit and wheat flours enriched with edible fish meal

**DOI:** 10.1002/fsn3.1919

**Published:** 2020-09-30

**Authors:** Adegoke H. Bakare, Abiodun A. Adeola, Ibijoke Otesile, Adewale O. Obadina, Wasiu A. Afolabi, Mojisola O. Adegunwa, Rachael A. Akerele, Olaoluwa O. Bamgbose, Emmanuel O. Alamu

**Affiliations:** ^1^ Department of Hospitality and Tourism Federal University of Agriculture Abeokuta Nigeria; ^2^ Institute of Food Security Environment Resources and Agricultural Research Federal University of Agriculture Abeokuta Nigeria; ^3^ Department of Food Science and Technology Federal University of Agriculture Abeokuta Nigeria; ^4^ Department of Nutrition and Dietetics Federal University of Agriculture Abeokuta Nigeria; ^5^ Department of Home Economics and Hotel Management Tai Solarin University of Education Ijebu Ode Nigeria; ^6^ Food and Nutrition Sciences Laboratory International Institute of Tropical Agriculture Southern Africa, Research and Administration Hub (SARAH) Campus Lusaka Zambia

**Keywords:** breadfruit flour, edible fish meal, optimization, product development, texture and biscuits

## Abstract

This study aimed to develop biscuits with improved nutritional contents using edible fish meal from catfish as the source of macro‐ and micronutrient enrichment while trying to reduce the input of wheat flour in biscuit‐making process. The biscuit was produced using edible fish meal (EFM: 0%–40%) from catfish, improved quality breadfruit (IQBF: 0%–60%), and wheat flours (WF: 0%–40%). Macro (crude protein, fat, fiber, ash, and carbohydrate)‐ and micro (calcium, magnesium, potassium, phosphorus, sodium, and iron)‐nutrient contents of the biscuit were determined. The color (lightness—L*, redness—a*, and yellowness—b*), texture (hardness, springiness, and adhesiveness), and sensory (taste, texture, and overall acceptability) attributes of the biscuits were assessed using standard methods. Model characteristics of the responses were profiled, and numerical optimization technique was used to predict combination/blends that produce biscuits with desired nutritional contents. Moisture, crude protein, fat, fiber, and ash values were in the range of 3.50%–5.57%, 3.06%–15.52%, 13.62%–26.00%, 0.31%–1.40%, and 1.98%–5.32%, respectively. The iron, calcium, and phosphorus contents of the biscuit ranged from 103.85 to 201.30 mg/100 g, 100 to 754 mg/100 g, and 8 mg/100 g to 304 mg/100 g, respectively. Interaction between the models for WF and EFM was significant and this significantly affected the L* (36.37–51.90) and adhesiveness (0.01–0.29) values for color and texture, respectively. Similar observations were also noticed for most of the nutrients. The quadratic models selected for the nutrients were all significant (*p* < .05) and the adjusted *R* ^2^ ranged from 0.61 to 0.84 and 0.59 to 0.97 for the macro‐ and micronutrients, respectively. In conclusion, a biscuit from IQBF, WF, and EFM of 61.33, 0.07, and 38.60 with protein, fat, ash, iron, and calcium contents of 10.41%, 17.59%, 2.05%, 120.52 mg/100 g, and 500.00 mg/100 g, respectively, was produced.

## Graphical abstract

This study addressed the concern about the alteration in baking, textural, and sensory qualities that occurred in biscuit when composite technology is used as a means of nutritional enrichment. Optimal modeling of these quality attributes provided baseline knowledge that may enable food product developers, food production managers, and other personnel involved in equipment design and research to explore the design space to produce nutritionally enriched biscuits with minimal quality defects

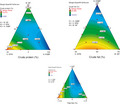

## 1 INTRODUCTION

Biscuit (Cookies) are a group of confectionery products made from flour, sugar, egg, and shortening. As convenient food, its inclusion in the diet of Nigerians has increased appreciably, particularly among children and during festivities. Its acceptability as snacks cut across cultural and sociodemographic boundaries. The low‐moisture nature of biscuit eases transportation difficulties and reduces potential health hazard from microbial contamination. Wheat flour has been a significant ingredient in conventional biscuit making. It was estimated that wheat imports might grow at 5 percent per annum, and the country could be importing as much as 10mmt per annum by 2030 (AEGIC, 2015). Adoption of composite flour technology might have significant saving on foreign exchange, more so when most of the biscuits that appealed to people of various age groups can be made with soft flours. The use of nonwheat adjuncts that can play complementary roles in reducing protein‐energy malnutrition and having potentials to alleviate associated social problems might be more promising options.

Fish is an essential source of high‐quality protein (Ohen & Abang, 2007), providing about 16% of animal protein consumed by the world's population. It provides 40% of the dietary intake of animal protein of the average Nigerian (Federal Department of Fisheries, 1997). Catfish is highly nourishing. It contains lysine as well as vitamin A that is necessary for healthy growth. It contains some quantities of calcium, phosphorus, fat, and other nutrients needed for human growth and health (FAO, 2003). Nigeria is one of the largest importers of fish in the developing world, importing about 600,000 metric ton annually to meet the country's high demand for fish (Olagunju et al., 2007). Updated official production statistics are unavailable, and production is presently estimated at 1.7 million metric tons of fish annually (Abba, 2012). Catfish farming is undertaken by a large number of people, especially the small‐scale farmers in Nigeria (Oladejo, 2010). The current motivation of small‐scale farmers in Nigeria needs to be encouraged and sustained. Most of these farmers are into mainly catfish production, and these are sold mostly in a raw state to middlemen for sale to domestic consumers. It is, therefore, necessary to develop value addition chain that would encourage increase production and industrial utilization. This can be done by developing an edible fish meal (EFM) from catfish and using it as a form of nutritional supplementation. EFM is the product obtained from the removing of water and oil from whole fish, thereby increasing the concentration of protein and other nutrients (Ibrahim, 2009). EFM provides the opportunity of utilizing other nutrients such as calcium that are available in the fish (Table 1).

**Table 1:** Experimental design

Experimental Runs	Experimental Design		
	IQBF	WF	EFM
1	80.00	20.00	0.00
2	73.16	0.00	26.84
3	60.00	0.00	40.00
4	60.00	40.00	0.00
5	73.21	13.46	13.33
6	86.53	6.79	6.68
7	60.00	0.00	40.00
8	80.00	0.00	20.00
9	80.00	20.00	0.00
10	60.00	40.00	0.00
11	100.00	0.00	0.00
12	100.00	0.00	0.00
13	66.59	26.91	6.49
14	60.00	20.41	19.59
15	66.31	7.05	26.64
16	60.00	20.41	19.59

Breadfruit (*Artocarpus communis* Forst) is a staple diet in many tropical countries. The tree fruits early between May and August, producing 50–200 fruits in a year. The mature fruit is round or ovoid, 15–20 cm in diameter, and weighs 2–10 kg on average. The fruit is produced mainly in Malaysia, the South Pacific Island, the Caribbean, and West Africa (Ragone, 2009). Total yearly production in Nigerian is about 10 million metric tons with potential to exceed 100 million metric tons with improved agricultural practice (Adewusi et al., 1995; Amusa et al., 2002). The economic utilization of Breadfruit has been limited by its poor storage properties which are about 1–3 days after harvest (Ragone, 2009). Conversion of breadfruit to the flour would provide a more stable storage form and also enhance its versatility (Morton, 1987; Oladunjoye et al., 2010). Although breadfruit is a perennial crop, it has relatively less competing for domestic uses and has a higher yield per tree per year that can be sustained for many years; an average‐sized tree reportedly produced 400–600 fruits per year (NTBG, 2009). Its horticultural features can also be explored at the household level to mitigate the effect of climate change in addition to its economic advantage. The fruit is highly perishable and has an undesirable fruity odor that impacts major organoleptic challenges to the acceptability of products made from it. Breadfruit is enzyme active even during processing to flour. Improvement in flour quality can be achieved by modifying processing procedure and reducing processing time.

Replacement of wheat flour with other adjuncts influences many functional properties of the dough and quality attributes of the resulting biscuits even when it is partial. Biscuits are grouped into various classes such as crackers, sweet biscuits, strongly sweet, semi‐sweet, wafers (Manley, 1983), each associated with peculiar quality characteristics, particularly texture. A major concern in applying composite flour technology to the development of bakery product is how to minimize the quality alteration, retain as much as practicable the quality attributes associated with an existing product, and make the product acceptable to the consumers. In cases where these become in impracticable, and products with unique quality have to be developed, it may be necessary to profile the quality attributes of such products. Development of nutritionally enriched biscuits may, therefore, require the profiling of its technological characteristics, particularly, its texture parameters and its relationship with sensorial texture to ascertain its suitability for industrial production and consumer's acceptance. This study evaluated the quality attributes of biscuit produced from an edible fish meal (EFM), improved quality breadfruit flour (IQBF), and wheat flour (WF). Specifically, it used D‐optimal design to model some of the nutritional quality, color, texture, spread ratio, and sensory qualities of the biscuit.

It also assessed the relationships between the sensory texture of the biscuits and some texture parameters of the biscuit. It described the quality attributes of biscuit that should be expected from the modeled responses.

## 2 MATERIALS AND METHODS

### 2.1 Source of materials

Seedless variety of freshly harvested 600 pieces of breadfruit (*Artocarpus communis* Forst) was purchased from a farm in Idiroko, Ogun State. Matured catfish (*Clarias gariepinus*) were obtained from a Fish farm in Ijebu‐Ode, Ogun State, Nigeria. Wheat flour (Honeywell brand) and other ingredients were gotten from retail markets in Abeokuta. Other ingredients were procured from retails markets within Abeokuta, Ogun State, Nigeria.

### 2.2 Methods

#### 2.2.1 Preparation of improved quality breadfruit flour (IQBF)

The IQBF was produced within 24 hr of harvest, using a modified method of Bakare et al. (2012). The matured fruits were thoroughly washed to remove adhering latex and dirt, peeled manually, washed, grated, bagged, dewatered, and pulverized. The whole batch of pulverized mash was dried using the flash dryer (Nobex Flash dryer, Nobex Technical Company Limited, Idimu Lagos, Nigeria) with the following conditions. The loading time was 10 min; the temperature in the tube and that of inlet air were 180°C and 200°C, respectively. The feeding characteristics were moisture content: 45%; mass density: 1,380 kg/m^3^; and feed rate: 820 kg/hr. The powder was allowed to settle and discharge at every 10 min. The dried breadfruit was milled using locally fabricated hammer mills, sieved (W.S. Tyler, 8570 Blvd, Mentor, OH, United States) through a 250‐μm mesh sieve, and sealed in polythene bags and used for analyses.

#### 2.2.2 Preparation of spice mixture

Formulated instant spice mixture (ISM) was prepared as described by Bakare et al. (2016) and added to confer flavor and functionality properties on the biscuit.

#### 2.2.3 Preparation of edible fish meal (EFM)

EFM was produced as described by Bakare et al. (2019), with some modifications. Catfish of consistent (About 500 g) weights was washed, eviscerated, steamed at 95°C for 25 min, and dried in forced convectional air dryer (Nexus, NX‐AF3100(2), Deekay Group (Nig.) Ltd, Nigeria) at 180°C for 45 min and pressed. The dried catfish was cooled, milled to powdery form using locally fabricated Hammer mill, sieved (W.S. Tyler, 8570 Blvd, Mentor, OH, United States) through a 250‐μm mesh sieve, packed in an airtight container, and stored for subsequent use.

#### 2.2.4 Biscuit production

The biscuit was produced based on an experimental (D‐optimal) design involving the use of IQBF, WF, and EFM as independent variables. The biscuit was produced as described by Manley (1983) and modified by Bakare et al. (2014). Percentage recipe formulation included the following: blends of flour (63.5), sugar (12.7), fat (15.9), invert syrup (6.35), sodium bicarbonate (0.5), baking powder (0.5), and ISM (0.6).

#### 2.2.5 Experimental design and optimization

The D‐optimal design used consisted of sixteen (16) experimental runs having five replications at the central point, which allows for estimation of pure error sum of squares. The coded and actual variables at the three levels (−1, 0, and +1) in which attributes of each of the factor {IQBF: (60.00, 80.00, and 100), WF (0.00, 20.00, and 40.00), and EFM (0.00, 20.00, and 40.00)} at high, central, and low levels were chosen based on preliminary experiments.

Fitness of each of the models was analyzed to identify the model that can best be used as a response predictor. The models were assessed for their adequacy for the experimental conditions and significant terms in each of the models were identified, and numeric optimization of the mixture blends was done based on set targets (Table 2).

**Table 2:** Optimization goals for process variables

Constraints	Goal	Lower limit	Upper limit
IQBF (%)	As in range	60	100
WF (%)	As in range	0	40
EDM (%)	As in range	0	40
Moisture content (%)	Minimize	3.50	5.57
Crude protein (%)	Maximize	3.06	15.52
Crude fat (%)	As in range	13.62	26.00
Crude fiber (%)	Maximize	0.31	1.40
Total ash (%)	Maximize	1.98	5.32
Total carbohydrate (%)	Is in range	51.58	75.56
Sodium (mg/100 g)	Minimize	305.25	401.30
Iron (mg/100 g)	Maximize	103.85	201.30
Calcium (mg/100 g)	Maximize	100.00	754.00
Magnesium (mg/100 g)	As in range	55.00	72.50
Potassium (mg/100 g)	As in range	377.50	582.00
Phosphorus (mg/100 g)	Maximize	38.00	304.00
L*	As in range	36.36	52.84
a*	As in range	3.42	9.80
b*	As in range	13.05	22.07
Hardness	As in range	48.23	2,118.4
Chewiness	As in range	0.09	1,111.15
Gumminess (N)	As in range	2.11	1608.33
Cohesiveness	As in range	0.054	0.78
Springiness	As in range	0.05	0.72
Stringiness	As in range	0.72	1.65
Deformation at peak	As in range	1.39	1.79
Adhesiveness	As in range	0.01	0.29
Energy to break	As in range	0.04	1.74
Appearance	Maximize	6.36	7.12
Texture	Maximize	5.56	6.88
Taste	Maximize	5.28	6.72
Color	Maximize	5.56	6.92
Overall acceptability	Maximize	5.48	6.88
Spread ratio	Is in range	7.94	11.29

Note

IQBF, WF, EFM = Improved quality breadfruit flour, wheat flour, and edible fish meal.

The measured responses were as follows: nutritional [proximate (moisture, crude protein, fat, fiber, total ash, and carbohydrate), micronutrients (sodium, iron, calcium, magnesium, potassium, and phosphorus) compositions, color (lightness, redness, and yellowness), instrumental texture (hardness, chewiness, gumminess, cohesiveness, springiness, stringiness, deformation at peak, adhesiveness, and energy to break), sensory (appearance, texture, taste, color, and overall acceptability) properties, and spread ratio].

#### 2.2.6 Analyses

All analyses were performed in triplicates. Moisture (AOAC method 950.46B), ash (AOAC method 920.153), protein (AOAC method 955.04), and lipid (AOAC method 991.36) contents were determined according to Association of Official Analytical Chemists procedures (AOAC, 2005). Total carbohydrate contents were calculated by difference using the following equation: % carbohydrates = 100% − (% moisture + % protein + % ash + % lipid), while energy values were determined following the formula: energy value (kcal/100 g) = 4 × protein (%) + 9 × lipid (%) + 4 × carbohydrate (%) as described by Bonfim et al. (2019). The crude fiber content of flour was determined by the trichloroacetic acid method as described by Entwistle and Hunter (1949). The mineral content of the biscuit samples was determined using the method described by Adeniji and Tenkouano (2008). In the analysis, 1 g of sample was weighed into a pyrex glass conical flask, and 10 ml of concentrated nitric acid was introduced into the flask with a straight pipette, and then, 5 ml of perchloric acid was also added. The mixture was heated until a clear digest was obtained then the digest was cooled to room temperature and diluted to 50 ml with distilled water. The diluent was filtered into a plastic vial for atomic absorbance spectrophotometer analysis. The phosphorus in the sample filtrate was determined by colorimetric method at 400 nm. Color of the biscuits was measured using a colorimeter (Konica Minolta CR‐210 chronometer) and recorded in the L*, a*, b* color system. The colorimeter was calibrated using a standard white plate. Samples were placed in the sample holder for measurement. Color values were recorded as L*(lightness), a* (redness), b* (yellowness) color system as described by Akissoe et al., (2003).

#### 2.2.7 Texture profile of biscuit

The texture of the biscuits was determined using the Universal testing machine (model M500‐100AT, Testometric, England). Compressive stress was applied to the samples to determine the behavior of the biscuits under a compressive load. A flat plunger with a 75 mm diameter was attached to the crosshead of the machine. Each biscuit was compressed uniaxially at a depth of 15 mm (30% strain) with a crosshead speed of 102 mm/minutes. Both load cell and strain gauges were connected to a data logging system to record the data. The stress–strain data were continuously logged into a computer, and the stress at failure was considered as the uniaxial compression strength (UCS) of biscuits. Specifically, the parameter measured were hardness, chewiness, gumminess, cohesiveness, springiness, stringiness, and force at the peak, deformation at the peak, energy to peak, adhesiveness, and energy to break.

#### 2.2.8 Physical properties

The spread ratio of the biscuit samples was determined, as described by Gaines (1991). Six biscuits edge to edge were used for the evaluation, and the average was noted. Spread ratio was calculated by dividing the diameter by thickness of the biscuits.

#### 2.2.9 Sensory evaluation

Sensory evaluation was conducted as described by Iwe (2002) by using quantitative acceptance to assess consumers liking for the biscuit. Thirty untrained panelists rated their liking or otherwise for the product from the blends on a nine‐point Hedonic scale (1 = disliked extremely as compared to reference sample “*R*,” and 9 = liked extremely as compared to “R”).

#### 2.2.10 Data/statistical analyses

Data were subjected to analysis of variance. Differences between mean values were separated using Duncan multiple range tests (Duncan, 1955). Pearson product–moment correlation coefficients were used to test the relationships between some of the variables. Independent *t* test was used for the validation experiment. Statistical analysis package for social science (version 23, IBM, Armonk, NY, USA) was used for these analyses.

Design expert software (version 7.00 Stat, Ease Inc., Minneapolis, MN, USA) was used for the experimental design and subsequent numerical optimization.

## 3 RESULTS AND DISCUSSION

### 3.1 Nutritional attributes

#### 3.1.1 Moisture and macronutrients content

The moisture content of food is one of the most important and widely used indices for determining the quality of dried processed foods. It is a measure of yield and quantity of food solids and can be a direct index of economic value, stability, and quality (WMC, K‐State, NCI, and NAEGA, 2008). The moisture content of biscuits samples (Table 2) ranged from 3.50% to 5.57%. Biscuit produced from 60% IQBF: 0% WF: 40% EFM had the lowest moisture content and that of 66.31% IQBF:7.05% WF:26.64% EFM had the highest. The moisture content values of the blends varied significantly (*p* < .05) from each other and are similar to values reported by Passos et al. (2013) for various types of biscuits brands.

Proteins are made up of amino acids. They are required for the synthesis of body protein and other critical nitrogen‐containing compounds (Roth, 2011). Proteins and other nitrogenous compounds are being degraded and resynthesized continuously. A continuous supply of dietary amino acids is therefore required to replace these losses, even after growth has ceased. Inadequate supply of dietary protein could be physically manifested in stunting, poor musculature, edema, thin and fragile hair, skin lesions in children while biochemical changes like low serum albumin and hormonal imbalances. Edema and loss of muscle mass and hair are the prominent signs in adults. Biscuit is diverse in terms of quality attributes, and this makes it to be suitable for nutritional enrichment and development of domestic agriculture production (Noorfarahzilah et al., 2014) through the use of composite flour technology.

The crude protein of biscuits samples (Table 2) ranged from 3.06% to 15.52%. The lowest crude protein (3.06%) value was obtained in the flour blends of 100% IQBF, 0% WF, and 0% EFM (experimental runs 11 and 12) while the highest (15.52%) was obtained with the flour blends of 60% IQBF, 0% WF, and 40% EFM (experimental runs 3 and 7). The biscuits from the flour blends varied significantly (*p* < .05) in their protein contents. The values obtained in this study were comparable to those reported by Norhayati et al. (2015) for commercial biscuits sold in Malaysia, Passos et al. (2013) for industrialized biscuits, Adeola and Ohizua (2018) for biscuits from composite flour of unripe cooking banana, pigeon pea, and sweet potato, and Bakare et al. (2014) for the breadfruit–wheat composite biscuit. However, the inclusion of EFM at between 20% to 40% level significantly increased the protein content of the biscuits to between 10.25% and 15.52%. An adequate supply of proteins in the daily diet is essential for healthy growth and development and the maintenance of health. Proteins build and repair body tissue play significant roles in regulating various body functions and provide energy if there are insufficient carbohydrate and fat in the diet (Roth, 2011). The size of the Nigerian biscuit segment has been estimated at N121 billion (US$617 million), having grown at a food sales cumulative annual growth rate of 16% in the past five years while annual production is estimated at 152,490 tons (KPMG, 2016). Modification of recipe formulation to accommodate EFM for biscuits might have an appreciable reduction in the use of wheat flour and even reduce the quantity of hydrogenated vegetable fat needed for the production of the biscuit.

Fat is necessary for steroids and hormones produced in the body and serves as solvents for hormones. It contains essential fatty acids and acts as carriers for fat‐soluble vitamins A, D, E, and K and has the highest caloric content compared to protein and carbohydrate. Thus, it provides relatively higher (9 calories per gram of fat) amount of energy (Roth, 2011). Fats are essential for the functioning and structure of body tissues. Extra fat is stored in adipose tissue and is burnt when the body has run out of carbohydrates. Deficiency symptoms of under consumption of fat beneath 10% of the total daily calorie requirement that have been reported include eczema (inflamed and scaly skin condition), retarded growth, and weight loss. On the other hand, excessive fat in the diet can lead to obesity or heart disease (Roth, 2011). Studies have also pointed to an association between high‐fat diets and cancers of the colon, breast, uterus, and prostate (Roth, 2011). Fat intake is not expected to be more than 30% of total calories (US Department of Agriculture, Center for Nutrition Policy and Promotions, 2005). In this study, the crude fat of biscuits samples (Table 2) ranged from 13.62% to 26.00%. Biscuit produced from 100% QBF:0% WF:4 0% EFM) had the lowest fat content and that of 66.31% IQBF:7.05% WF:26.64% EFM had the highest. There were significant (*p* < .05) differences in crude fat contents of the biscuit blends. The result suggested the need to defat the EFM further or modify the recipe to reduce the fat content of the biscuit. An individual's average daily energy requirement is the total number of calories needed in 24 hr. Energy requirements of people differ, depending on the basal metabolic rate (BMR) and activities. BMR is the daily rate of energy metabolism an individual needs to sustain in order to preserve the integrity of vital functions (Henry, 2005; Hulbert & Else, 2004; Mitchell, 1964). Less active children, and female and male adults (Above 50 years) require 1,000, 1,600, and 2,000 calories (US Department of Agriculture, Center for Nutrition Policy and Promotions, 2005).

The fiber content of the biscuits (Table 2) ranged from 0.31% to 1.40%. The lowest crude fiber (0.31%) value was observed in the blends of 60% IQBF, 0% WF, and 40% EFM (experimental runs 3 and 7), while the highest (1.40%) was in 100% IQBF, 0% WF, and 0% EFM (experimental runs 11 and 12). The crude fiber contents of the samples were significantly (*p* < .05) different from each other. These values were relatively lower than values reported in the previous study for biscuits produced from fermented breadfruit flour prepared by a conventional process (Bakare et al., 2014). The method of preparation of IQBF used for this study yielded breadfruit flour with reduced crude fiber content (2.49%) compared to the crude fiber content range of 6.32%–9.04% reported for conventional breadfruit flour (Bakare et al., 2012). The implications of dietary fiber have been documented and cited in the previous publication (Bakare et al., 2012). Consumption of dietary fiber is, therefore, necessary for the prevention of constipation, hemorrhoids, and diverticular disease by softening and increasing the size of the stool. The optimal recommendation for dietary fiber intake is 20–35 g/day. However, consumption of too much fiber can induce discomfort, flatulence (abdominal gas), and diarrhea. It also could obstruct the gastrointestinal tract if intake exceeds 50 grams per day (Roth, 2011). Insoluble fiber contains binders in the form of phytic acid or phytate, which can prevent the absorption of minerals such as calcium, iron, zinc, and magnesium. Preponderantly, there is little no evidence to assert that the traditional diet of Africans and specifically Nigerians is deficient in dietary fiber despite the dietary transition occasioned by changing lifestyle so excess intake needs to be avoided.

The ash content provides insights into the mineral quality of the biscuit. The ash content of the biscuits (Table 2) samples ranged from 1.98% to 5.32%. Biscuit produced from 80% IQBF: 20% WF:0% EFM had the lowest ash content, and that of 66.31% IQBF:7.05% WF:26.64% EFM had the highest. The ash contents of the biscuit increased significantly across the blends. Higher ash content values of the biscuit produced from the blends containing EFM suggested an improvement in the micronutrient quality of those blends. The carbohydrate content of the biscuits samples ranged from 51.58% to 75.56%. Experimental runs 11 and 12 (100% IQBF, 0% WF, and 0% EFM) had the highest value (75.56%) while experimental runs 15 (66.31% IQBF, 7.05% WF, and 26.64% EFM) had the least value (51.58%).

The total energy content of the biscuit is presented in Table 2. The estimated energy content of a food substance is a function of the total protein, fat, and carbohydrates present in the biscuit. The total energy value of biscuits samples ranged from 4,556.51 to 5,247.45 kCal/kg. Experimental runs 11 and 12 (100% IQBF, 0% WF, and 0% EFM) had the least value, while experimental runs 3 and 7 (60% IQBF, 0% WF, and 40% EFM) had the highest value. The energy contents of the biscuit samples varied significantly (*p* < .05) between the samples.

##### Mineral contents

The mineral contents of the biscuit presented in Table 2 included some of the micro (Trace)‐ and macrominerals. Macrominerals (sodium, calcium, magnesium, potassium, and phosphorus) are required in amounts greater than 100 mg a day while the microminerals (iron) are needed in amounts smaller than 100 mg a day.

The sodium content of biscuit samples ranged from 305.25 to 401.00 mg/100 g. The least sodium (305.25 mg/100 g) was observed in the blend containing 86.53% IQBF, 6.79% WF, and 6.79% EFM (experimental runs 6), while the highest value (401.00 mg/100 g) was in a blend containing 66.31% IQBF, 7.05% WF, and 26.64% EFM (experimental runs 15). The sodium contents of the samples were significantly (*p* < .05) different from each other. However, biscuits sample produced from 73.21% IQBF, 13.46% WF, and 13.33% EFM and 80% IQBF, 0% WF, and 20% EFM (experimental runs 5 and 8) were not significantly (*p* > .05) different from each other but are different from other samples. Generally, exogenous sources of sodium into the biscuit could have been from the aerating agents while the endogenous sources are usually from the primary ingredients such as the EFM, flour blends, and bakery fat. Sodium is needed for the maintenance of fluid balance, the transmission of nerve impulses, acid–base balance, and regulation of muscle and nerve activities but excess intake of sodium is associated with cardiovascular conditions such as hypertension and congestive heart failure (Roth, 2011). The dietary reference intake (DRI) for sodium has been established at between 1,200 and 1,500 mg/day for adults (Roth, 2011). Therefore, the values observed in this study are within safe limits.

Iron is a component of hemoglobin and myoglobin, and it is needed for the delivery of oxygen to body tissues and cells. It is also needed by enzymes that are involved in the making of amino acids, hormones, and neurotransmitters (Agarwal, 2001; Ward et al., 2014). The recommended dietary reference intake of 10 mg and 15 mg for men and women above 11 years of age, respectively, can be easily met by the consumption of 100 g of this biscuit. The iron content of biscuit (Table 2) samples ranged from 103.85 to 210.30 mg/100 g. The least iron content (103.85 mg/100 g) was obtained from flour blends of 86.53% IQBF, 6.79% WF, and 6.68% EFM (experimental runs 6) while the highest value (210.30 mg/100 g) was recorded with the flour blends of 100% IQBF, 0% WF, and 0% EFM (experimental runs 11 and 12). The iron contents were significantly (*p* < .05) different from each other. Moreover, the result suggested that the IQBF being of fruit origin is a good source of iron despite the limitation associated with nonheme iron.

Calcium, in combination with phosphorus, is a component of bones and teeth, giving them strength and hardness. Calcium is also needed for normal nerve and muscle action, blood clotting, heart function, and cell metabolism. The calcium content of biscuits samples (Table 2) ranged from 100 to 754 mg/100 g. Lowest calcium content (100 mg/100 g) was observed in flour blends containing 100% IQBF, 0% WF, and 0% EFM (experimental runs 11 and 12) while the highest (754 mg/100 g) was in blends containing 60% IQBF, 0% WF, and 40% EFM (experimental runs 3 and 7). There were significant (*p* < .05) differences in the calcium content of the biscuit samples. However, some of the biscuits samples were not significantly (*p* > .05) different from each other. The estimated requirement for calcium is expressed as an adequate intake (AI) level. Calcium requirements vary between age group, gender, and peculiarity of needs. The recommended AI for children of 4–18 years and adults are between 800 to 1300 mg/day and 1000 mg to 1200 mg/day, respectively (National Academies of Sciences, 2006). The values obtained in this study for a biscuit from blends containing EFM indicated that 40% to 80% of these requirements could be met by the consumption of 100 g of this biscuit.

Magnesium is vital to both hard and soft body tissues. It is essential for metabolism and regulates nerve and muscle function, including the heart, and plays a role in the blood‐clotting process (Roth, 2011). Though rare, the deficiency symptoms included nausea and mental, emotional, and muscular disorders. The magnesium content of the biscuits samples ranged from 57 to 72.50 mg/100 g There was significant (*p* < .05) difference in the level of magnesium of the biscuit samples. The recommended daily intake (RDI) of magnesium ranges from 80 to 130 mg for children of 1 to 8 years and 240 to 420 mg for adults of 9 to 70 years (Roth, 2011). Thus, 100 g of the biscuits with the lowest value of magnesium will supply 43.8%–71.25% of RDI for children.

Potassium is an electrolyte that is predominant in intracellular fluid. Like sodium, it is essential for fluid balance and osmosis. It is also necessary for the transmission of nerve impulses and for muscle contractions. Some of its deficiency symptoms included diarrhea, vomiting, diabetic acidosis, and severe malnutrition. Additional symptoms are nausea, anorexia, fatigue, muscle weakness, and heart abnormalities. The potassium content of the biscuits samples ranged from 377.50 to 582 mg/100 g (Table 3). The least potassium content (377.50 mg/100 g) was obtained from flour blends of 86.53% IQBF, 6.79% WF, and 6.68% EFM (experimental runs 6) while the highest value (582 mg/100 g) was recorded at the flour blends of 100% IQBF, 0% WF, and 0% EFM (experimental runs 11 and 12). The potassium content of the biscuit samples was significantly (*p* < .05) different from each other.

**Table 3:** Proximate and mineral composition of biscuits

	Macronutrient							Micronutrient						
ER	MC (%)	Crude protein (%)	Crude fat (%)	Crude fiber (%)	Total ash (%)	CHO (%)	TE	Sodium (mg/100 g)	Iron (mg/100 g)	Calcium (mg/100 g)	Magnesium (mg/100 g)	Potassium (mg/100 g)	Phosphorus (mg/100 g)
1	5.50 ± 0.00^e^	4.46 ± 0.05^b^	17.06 ± 0.08^c^	1.32 ± 0.02^g^	1.98 ± 0.08^a^	69.70 ± 0.03^j^	4,758.74 ± 12.47^ab^	314.90 ± 2.12^c^	119.35 ± 1.34^c^	395.00 ± 14.14^c^	57.00 ± 5.66^ab^	435.00 ± 2.83^b^	196.00 ± 1.41^d^
2	5.00 ± 0.00^d^	5.26 ± 0.10^d^	**21**.42 ± 0.11^g^	1.07 ± 0.02^f^	2.39 ± 0.04^b^	64.87 ± 0.04^g^	4,786.70 ± 37.88^ab^	370.85 ± 2.19^g^	118.65 ± 0.21^c^	480.00 ± 7.07^d^	66.50 ± 0.71^bc^	485.00±0.00^cd^	229.50 ± 10.61^f^
3	3.50 ± 0.02^a^	15.52 ± 0.06^k^	22.50 ± 0.04^h^	0.31 ± 0.04^a^	5.32 ± 0.04^h^	52.87 ± 0.06^b^	5,247.45 ± 6.19^c^	311.10 ± 0.42^b^	108.50 ± 2.12^ab^	754.00 ± 16.97^f^	72.50 ± 2.12^c^	449.00 ± 15.56^dc^	304.00 ± 9.90^g^
4	3.52 ± 0.05^a^	12.59 ± 0.05^j^	20.74 ± 0.06^f^	0.45 ± 0.02^b^	4.58 ± 0.06^fg^	58.15 ± 0.21^c^	5,037.77 ± 9.50^bc^	346.05 ± 1.20^f^	140.60 ± 0.85^e^	404.50 ± 9.19^c^	67.00 ± 1.41^bc^	511.50 ± 31.82^d^	47.00 ± 5.66^a^
5	5.04 ± 0.06^d^	10.86 ± 0.04^h^	19.52 ± 0.02^e^	0.80 ± 0.03^d^	3.81 ± 0.04^e^	59.98 ± 0.03^d^	4,957.42 ± 37.21^abc^	337.50 ± 1.98^e^	129.65 ± 1.20^d^	555.00 ± 16.97^e^	59.00 ± 2.83^ab^	447.00 ± 33.94^bc^	178.50 ± 3.54^c^
6	4.48 ± 0.03^c^	6.04 ± 0.09^e^	16.45 ± 0.08^b^	1.04 ± 0.02^f^	2.90 ± 0.06^d^	69.10 ± 0.24^i^	4,760.54 ± 39.39^ab^	305.25 ± 1.20^a^	103.85 ± 0.35^a^	200.00 ± 15.56^b^	60.00 ± 4.24^ab^	377.50 ± 9.19^a^	62.00 ± 0.00^b^
7	3.50 ± 0.02^a^	15.52 ± 0.06^k^	22.50 ± 0.04^h^	0.31 ± 0.04^a^	5.32 ± 0.04^h^	52.87 ± 0.06^b^	5,247.45 ± 6.19^c^	311.10 ± 0.42^b^	108.50 ± 2.12^ab^	754.00 ± 16.97^f^	72.50 ± 2.12^c^	449.00 ± 15.56^bc^	304.00 ± 9.90^g^
8	4.45 ± 0.08^c^	10.25 ± 0.01^j^	16.47 ± 0.04^b^	0.82 ± 0.02^d^	4.37 ± 0.04^f^	63.66 ± 0.05^f^	4,778.25 ± 34.90^ab^	340.15 ± 0.35^e^	135.10 ± 0.85^de^	559.50 ± 3.54^e^	58.50 ± 7.78^ab^	479.50 ± 2.12^cd^	180.50 ± 2.12^c^
9	5.50 ± 0.00^e^	4.46 ± 0.05^e^	17.06 ± 0.08^c^	1.32 ± 0.02^g^	1.98 ± 0.08^a^	69.70 ± 0.03^j^	4,758.74 ± 12.47^ab^	314.90 ± 2.12^c^	119.35 ± 1.34^c^	395.00 ± 14.14^e^	57.00 ± 5.66^ab^	435.00 ± 2.83^b^	196.00 ± 1.41^d^
10	3.52 ± 0.05^a^	12.59 ± 0.05^j^	20.74 ± 0.06^f^	0.45 ± 0.02^b^	4.58 ± 0.06^fg^	58.15 ± 0.21^c^	5,037.77 ± 9.50^bc^	346.05 ± 1.20^f^	140.60 ± 0.85^e^	404.50 ± 9.19^c^	67.00 ± 1.41^bc^	511.50 ± 31.82^d^	47.00 ± 5.66^a^
11	3.90 ± 0.01^b^	3.06 ± 0.04^a^	13.62 ± 0.13^a^	1.40 ± 0.04^h^	2.48 ± 0.04^bc^	75.56 ± 0.17^k^	4,556.51 ± 40.46^a^	329.95 ± 1.34^d^	201.30 ± 5.37^f^	100.00 ± 5.66^a^	59.00 ± 2.83^ab^	582.00 ± 7.07^e^	38.00 ± 4.24^a^
12	3.90 ± 0.01^b^	3.06 ± 0.04^a^	13.62 ± 0.13^a^	1.40 ± 0.04^h^	2.48 ± 0.04^bc^	75.56 ± 0.17^k^	4,556.51 ± 40.46^a^	329.95 ± 1.34^d^	201.30 ± 5.37^f^	100.00 ± 5.66^a^	59.00 ± 2.83^ab^	582.00 ± 7.07^e^	38.00 ± 4.24^a^
13	3.98 ± 0.04^b^	6.38 ± 0.06^f^	19.00 ± 0.00^d^	1.02 ± 0.00^f^	2.47 ± 0.02^bc^	67.16 ± 0.04^h^	4,929.22 ± 4.73^abc^	311.25 ± 0.21^b^	108.55 ± 5.73^ab^	186.00 ± 2.83^b^	55.00 ± 1.41^a^	395.00 ± 11.31^a^	72.00 ± 4.24^b^
14	5.46 ± 0.06^e^	4.99 ± 0.04^c^	23.97 ± 0.05^i^	0.90 ± 0.01^e^	2.67 ± 0.13^cd^	62.01 ± 0.27^e^	5,111.30 ± 7.58^bc^	308.45 ± 2.47^ab^	120.35 ± 1.06^c^	393.00 ± 7.07^c^	61.50 ± 6.36^ab^	479.50 ± 3.54^cd^	205.00 ± 9.90^de^
15	5.57 ± 0.13^e^	11.78 ± 0.33^i^	26.00 ± 0.05^j^	0.59 ± 0.04^c^	4.75 ± 0.34^g^	51.58 ± 0.47^a^	4,788.27 ± 694.86^ab^	401.30 ± 0.57^h^	110.50 ± 2.12^b^	458.50 ± 4.96^d^	60.00 ± 5.66^ab^	462.00 ± 8.49^bc^	215.00 ± 5.66^e^
16	5.46 ± 0.06^e^	4.99 ± 0.04^c^	23.97 ± 0.05^i^	0.90 ± 0.01^e^	2.67 ± 0.13^cd^	62.01 ± 0.27^e^	5,111.30 ± 7.58^bc^	308.45 ± 2.47^ab^	120.35 ± 1.06^c^	393.00 ± 7.07^c^	61.50 ± 6.36^ab^	479.50 ± 3.54^cd^	205.00 ± 9.90^de^

Note

Mean values with different superscripts within the column are significantly different at *p* < 0–05.

CHO, total carbohydrate; MC, moisture content; TE, total energy (Kcal/Kg).

Phosphorus, in combination with calcium, is necessary for strong, rigid bones and teeth formation. It is important in the metabolism of carbohydrates, fats, and proteins. As a constituent of all body cells, it is necessary for a proper acid–base balance of the blood and is essential for the effective action of several B vitamins. The recommended estimated average requirements (EAR) is between 380 and 1,055 mg for children, the upper range being for children between the ages of 9 to 18 years. The phosphorus content of the biscuits samples (Table 3) ranged from 38 to 304 mg/100 g. The least phosphorus content (38 mg/100 g) was observed in 100% IQBF, 0% WF, and 0% EFM (experimental runs 11 and 12) while the highest (304 mg/100 g) was in 60% IQBF, 0% WF, and 40% EFM (experimental runs 3 and 7). There were significant (*p* < .05) differences in the phosphorus content of the biscuit sample. Phosphorus is found in many foods, and its deficiency is therefore rare.

#### 3.1.2 Color

A combination of the color index, including, L* (degree of lightness), a* (degree of redness) and b* (degree of yellowness) that were determined (Table 4) showed that the L* value of biscuit samples ranged from 36.37 to 51.90. The least value (36.37) was observed in 60% IQBF, 40% WF, and 0% EFM (experimental runs 4 and 10), while the highest value (51.90) was observed in 86.53% IQBF, 6.79% WF, and 6.68% EFM (experimental runs 6). The a* value of biscuit samples ranged from 3.52 to 9.80. The least value (3.52) was obtained from the flour blends of 60% IQBF, 20.41% WF, and 19.59% EFM (experimental runs 16) while the highest value (9.80) was recorded at the flour blends of 60% IQBF, 40% WF, and 0% EFM (experimental runs 4 and 10). The yellowness (b*) value of biscuit samples ranged from 13.06 to 21.24. The least value (13.06) was obtained from the flour blends of 60% IQBF, 20.41% WF, and 19.59% EFM (experimental runs 16) while the highest value (21.24) was recorded at the flour blends of 80% IQBF, 20% WF, and 0% EFM (experimental runs 9).

**Table 4:** Color and texture properties of biscuits

ER	Color Attributes			Texture Attributes								
	L*	a*	b*	Hardness (N)	Chewiness (N)	Gumminess (N)	Cohesiveness	Springiness	Stringiness	Adhesiveness (N.s)	Energy to Peak (N.m)	Def.@ Peak (mm)
1	50.25 ± 0.01^b^	4.04 ± 0.01^ab^	15.19 ± 0.34^abc^	1,726.47 ± 372.87^cd^	520.37 ± 280.00^b^	1,147.53 ± 466.04^ab^	0.66 ± 0.24^ab^	0.43 ± 0.09^bc^	1.07 ± 0.08^c^	0.18 ± 0.03	1.23 ± 0.27^ab^	1.74 ± 0.08^b^
2	46.12 ± 5.85^ab^	6.16 ± 3.01^abcd^	17.58 ± 3.02^bcd^	1,285.20 ± 61.17^bcd^	265.98 ± 146.98^bc^	749.52 ± 345.91^bc^	0.59 ± 0.02^abc^	0.32 ± 0.00^cd^	1.16 ± 0.22^bc^	0.02 ± 0.02	0.68 ± 0.27^bcd^	1.68 ± 0.03^b^
3	42.00 ± 0.03^ab^	8.29 ± 0.01^cd^	19.71 ± 0.01^de^	1,471.17 ± 469.73^cd^	259.89 ± 151.95^bc^	807.96 ± 278.95^bc^	0.55 ± 0.01^abc^	0.31 ± 0.07^cd^	1.21 ± 0.09^bc^	0.26 ± 0.32	0.89 ± 0.41^bc^	1.71 ± 0.03^b^
4	36.37 ± 0.02^a^	9.80 ± 0.00^c^	15.80 ± 0.25^abc^	2,116.80 ± 937.93^d^	551.00 ± 261.95^b^	1,065.04 ± 444.56^b^	0.51 ± 0.08^bc^	0.51 ± 0.03^b^	0.96 ± 0.11^cd^	0.29 ± 0.13	1.58 ± 0.83^a^	1.74 ± 0.01^b^
5	44.46 ± 11.45^ab^	7.50 ± 3.25^cd^	17.13 ± 1.97^bcd^	249.57 ± 104.77^a^	41.33 ± 24.37^c^	97.33 ± 30.99^d^	0.42 ± 0.12^c^	0.43 ± 0.21^bc^	0.97 ± 0.34^cd^	.	0.16 ± 0.06^d^	1.68 ± 0.03^b^
6	51.90 ± 0.92^b^	4.95 ± 0.00^abc^	17.90 ± 0.80^bcde^	1,238.80 ± 314.66^bc^	228.56 ± 80.63^c^	786.89 ± 231.91^bc^	0.63 ± 0.03^abc^	0.29 ± 0.06^cd^	1.22 ± 0.03^bc^	0.03 ± 0.01	0.66 ± 0.17^bcd^	1.70 ± 0.03^b^
7	52.52 ± 3.87^b^	4.09 ± 0.97^ab^	15.64 ± 0.15^abc^	1,471.17 ± 469.73^cd^	259.89 ± 151.95^bc^	807.96 ± 278.95^bc^	0.55 ± 0.01^abc^	0.31 ± 0.07^cd^	1.21 ± 0.09^bc^	0.26 ± 0.32	0.89 ± 0.41^bc^	1.71 ± 0.03^b^
8	49.24 ± 4.65^b^	5.68 ± 1.29^abc^	18.43 ± 3.97^cde^	282.19 ± 150.40^a^	5.33 ± 3.04^c^	56.17 ± 38.06^d^	0.20 ± 0.04^d^	0.11 ± 0.06^ef^	1.43 ± 0.07^ab^	.	0.16 ± 0.07^d^	1.79 ± 0.03^b^
9	45.96 ± 0.01^ab^	6.58 ± 0.02^abcd^	21.24 ± 0.01^e^	1,726.47 ± 372.87^cd^	520.37 ± 280.90^b^	1,147.53 ± 466.04^ab^	0.66 ± 0.24^ab^	0.43 ± 0.09^bc^	1.07 ± 0.08^c^	0.18 ± 0.03	1.23 ± 0.27^ab^	1.74 ± 0.08^b^
10	36.37 ± 0.02^a^	9.80 ± 0.00^d^	15.63 ± 0.01^abc^	2,116.80 ± 937.93^d^	551.00 ± 261.95^b^	1,065.04 ± 444.56^b^	0.51 ± 0.08^bc^	0.51 ± 0.03^b^	0.96 ± 0.11^cd^	0.29 ± 0.13	1.58 ± 0.83^a^	1.74 ± 0.01^b^
11	42.51 ± 8.67^ab^	7.12 ± 3.80^abcd^	14.98 ± 0.93^abc^	576.17 ± 107.99^ab^	84.45 ± 49.54^c^	367.08 ± 1.70^cd^	0.65 ± 0.12^abc^	0.23 ± 0.13^de^	1.17 ± 0.20^bc^	0.03 ± 0.10	0.30 ± 0.06^cd^	1.73 ± 0.06^b^
12	48.68 ± 0.00^b^	4.44 ± 0.00^ab^	14.35 ± 0.01^ab^	576.17 ± 107.99^ab^	84.45 ± 49.54^c^	367.08 ± 1.70^cd^	0.65 ± 0.12^abc^	0.23 ± 0.13^de^	1.17 ± 0.20^bc^	0.03 ± 0.10	0.30 ± 0.06^cd^	1.73 ± 0.06^b^
13	50.25 ± 0.01^b^	4.04 ± 0.01^ab^	15.10 ± 0.21^abc^	48.23 ± 11.43^a^	0.09 ± 0.07^c^	2.11 ± 2.16^d^	0.05 ± 0.06^d^	0.05 ± 0.02^f^	1.65 ± 0.13^a^	0.01 ± 0.10	0.03 ± 0.01^d^	1.39 ± 0.32^a^
14	51.40 ± 1.63^b^	4.63 ± 0.81^abc^	16.98 ± 2.18^bcd^	180.28 ± 107.92^a^	81.90 ± 61.81^c^	115.84 ± 82.32^d^	0.60 ± 0.14^abc^	0.72 ± 0.09^a^	0.72 ± 0.12^d^	.	0.13 ± 0.07^d^	1.65 ± 0.08^b^
15	51.80 ± 0.78^b^	5.06 ± 0.16^bcd^	17.86 ± 0.74^bcde^	2,118.40 ± 514.25^d^	1,111.15 ± 57.35^a^	1,608.33 ± 150.21^a^	0.78 ± 0.15^a^	0.69 ± 0.03^a^	0.94 ± 0.12^cd^	0.23 ± 0.03	1.74 ± 0.43^a^	1.79 ± 0.08^b^
16	49.99 ± 3.86^b^	3.52 ± 0.67^a^	13.06 ± 0.37^a^	180.28 ± 107.92^a^	81.90 ± 61.81^c^	115.84 ± 82.32^d^	0.60 ± 0.14^abc^	0.72 ± 0.09^a^	0.72 ± 0.12^d^		0.13 ± 0.07^d^	1.65 ± 0.08^b^

Note

Mean values with different superscripts within the column are significantly different at *p* < 0–05.

#### 3.1.3 Texture profiling

The hardness value of biscuits samples (Table 3) ranged from 48.23 to 2118.40N. Experimental runs 13 (66.59% IQBF, 26.91% WF, and 6.49% edible EFM) had the least value while experimental runs 15 (66.31% IQBF, 7.05% WF, and 26.64% edible EFM) had the highest value. The hardness values of the biscuits were significantly (*p* < .05) different from each other. The hardness value is the peak force that occurs during the first compression of the two‐cycle imitative tests which attempt to simulate the conditions to which the material is subjected to in the mouth. Hardness, in this case, is an indication of the force required to compress food between the molars (Rosenthal, 1999; Scott‐Blair, 1958). The lower the hardness values, the softer the biscuit. The values for hardness recorded in this study were higher than values reported for Maria cookies (Pereira^1^ et al., 2013).

The study was unable to establish a significant relationship between most of the texture (hardness chewiness, gumminess, cohesiveness, springiness, and stringiness) parameters and the composite (IQBF, WF, and EFM) blends. Studies have reported a strong correlation between instrumental and sensory measures of hardness (Campbell et al., 2016). The significant positive relationship (*n* = 16, *r* = 0.508 *p* = .44) between the hardness values and extent of deformation at the peak of the test indicated that the more the force applied to overcome the hardness, the more would be the extent of deformation. This is to be expected since biscuit of this type that is produced from short dough is not expected to be crispy, snappy but crunchy and possesses less resistance to deformation or effort to fracture. The oral processing of dry crunchy biscuits has reduced oral processing and leads to a reduction in the amplitude of jaw movement in both vertical and medial–lateral planes which are the opposite of the structural characteristics associated with the hardness of dried nuts or seeds (Çakir et al., 2012).

Chewiness value of biscuits samples (Table 5) ranged from 0.09 to 1,111.15N. Experimental runs 13 (66.59% IQBF, 26.91% WF, and 6.49% edible EFM) had the least value while experimental runs 15 (66.31% IQBF, 7.05% WF, and 26.64% edible EFM) had the highest value. It is the energy required to chew solid food until it is ready for swallowing. It is sometimes estimated as the product of hardness, cohesiveness, and elasticity (Rosenthal, 1999). Crunchy biscuit of this type should have reduced muscle activity during chewing and reduction in the number of chews required preparing the biscuit for swallowing. There were significant positive relationships between chewiness and cohesiveness ((*n* = 16, *r* = 0.525 *p *= .037) and also between chewiness and adhesiveness (*n* = 16, *r* = 0.629 *p *= .029).

**Table 5:** Relationships between the composite blends and texture parameters of the biscuit

S/N	1	2	3	4	5	6	7	8	9	10
1	IQBF									
2	WF	−0.475								
3	EFM	−0.505*	−0.519*							
4	Hardness (N)	−0.223	0.249	−0.028						
5	Chewiness (N)	−0.243	0.252	−0.012	0.863**					
6	Gumminess (N)	−0.136	0.148	−0.013	0.965**	0.932**				
7	Cohesiveness	0.189	−0.189	0.001	0.485	0.525*	0.594*			
8	Springiness	−0.491	0.398	0.086	0.274	0.493	0.331	0.579*		
9	Stringiness	0.312	−0.320	0.010	−0.178	−0.310	−0.204	−0.641**	−0.941**	
10	Adhesiveness (N.s)	−0.724**	0.408	0.276	0.823**	0.629*	0.692*	0.180	0.692*	−0.596*

** Correlation is significant at the 0.01 level (2‐tailed).

* Correlation is significant at the 0.05 level (2‐tailed).

The gumminess value of biscuits samples (Table 4) ranged from 2.11 to 1608.33N. Experimental runs 13 (66.59% IQBF, 26.91% WF, and 6.49% edible EFM) had the least value while experimental runs 15 (66.31% IQBF, 7.05% WF, and 26.64% edible EFM) had the highest value. However, the gumminess of the biscuits was significantly (*p* < .05) different from each other. Gumminess is conceived as the energy required to disintegrate a semisolid food to make it ready for swallowing ((Trinh & Glasgow, 2012). Gumminess may not be mutually exclusive with chewiness since crunchy biscuit; a solid product with chewy textural characteristics upon mastication with saliva may become adhere to the teeth and become gummy. This may explain the significant relationships between gumminess and chewiness (*n* = 16, *r* = 0.932, *p* = .000), cohesiveness (*n* = 16, *r* = 0.594 *p* = .015), and adhesiveness (*n* = 12, *r* = 0.692, *p* = .013), respectively (Table 4).

Cohesiveness reflects the strength of the internal bonds binding the food particles together and suggested how well the biscuit withstands a second deformation relative to its resistance under the first deformation. The higher the cohesion value, the greater the ability of the biscuit to break when subjected to stress. Similar values were observed for a certain type of cookies (Pereira^1^ et al., 2013). The cohesiveness value of the biscuits samples ranged from 0.05 to 0.78. Experimental runs 13 (66.59% IQBF, 26.91% WF, and 6.49% edible EFM) had the least value while experimental runs 15 (66.31% IQBF, 7.05% WF, and 26.64% edible EFM) had the highest value. The blends were significantly (*p* < .05) different from each other. Significant relationships were observed (Table 5) between cohesiveness and chewiness (*n* = 16, *r* = 0.525 *p *= .037), gumminess (*n* = 16, *r* = 0.594 *p* = 0 0.015), springiness (*n* = 16, *r* = 0 0.579 *p* = .019), and stringiness (*n* = 12, *r* = −0.641 *p* = .007), respectively.

Springiness is measured several ways, but most typically, by the distance of the detected height (hardness) during the second compression divided by the original compression distance. It organoleptically depicts how well a product physically springs back after it has been deformed during the first compression and has been allowed to wait for the target wait time between strokes. The springiness value of biscuits samples (Table 4) ranged from 0.05 to 0.72. Experimental runs 13 (66.59% IQBF, 26.91% WF, and 6.49% edible EFM) had the least value while experimental runs 14 and 16 (60% IQBF, 20.41% WF, and 19.59% edible EFM) had the highest value. Significant relationships (Table 4) existed between springiness and adhesiveness (*n* = 12, *r* = 0.692 *p* = 0 0.013); springiness is synonymous with elasticity in some products. The springiness values observed in this study is higher than the values reported for some cookies (Pereira^1^ et al., 2013).

**Figure 1:**
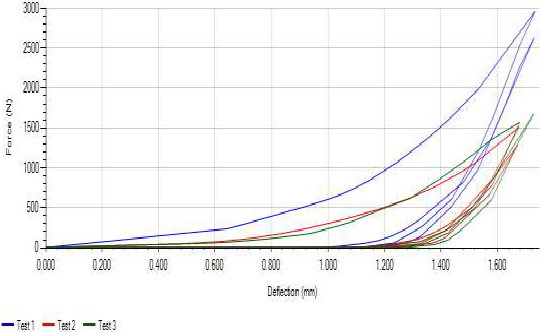
Typical curve of the Texture Profile of the Biscuit

The adhesiveness value of biscuits samples (Table 4) ranged from 0.01 to 0.29. Experimental runs 13 (66.59% IQBF, 26.91% WF, and 6.49% edible EFM) had the least value while Experimental runs 4 and 10 (60% IQBF, 0% WF, and 40% edible EFM) had the highest value. Adhesiveness is an indication of the extent of the stickiness of products. Work required overcoming the sticky forces between the sample and the probe (Trinh & Glasgow, 2012). Adhesion is measured as the negative work between the two cycles; however, in many instances, the product has stuck to the probe and does not separate when the highest point between the two cycles is just back to the original product height. There was a significant negative relationship between adhesiveness and IQBF (*n* = 16, *r* = −0.724 *p* = 0 0.008), hardness, chewiness, gumminess, springiness, and stringiness (Table 4) The typical curve for the texture profile is illustrated in Figure 1. The biscuits that are excessively adhesive would require more jaw movement in all three planes of movement during oral processing and more muscle activity even at equal compressive hardness (Çakir et al., 2012).

#### 3.1.4 Spread ratio

The values of the spread ratio of biscuits (Table 5) ranged from 9.43 to 11.29. The least value (9.43) was obtained from the flour blends of 80% IQBF, 0% WF, and 20% EFM (experimental runs 8) while the highest value (11.29) was recorded at the flour blends of 60% IQBF, 0% WF, and 40% edible EFM (experimental runs 3). However, the spread ratio of the biscuits was not significantly (*p* < .05) different from each other. Spread ratio is an important quality attribute of biscuit because of its relationship with texture, grain finesse, bite, and overall mouthfeel of the biscuits (Jothi et al., 2014).

#### 3.1.5 Sensory quality

The sensory attributes of the biscuit are presented in Table 5. The values for appearance ranged from 6.36 to 7.12, with blend experimental runs number 3 and 8 having the lowest and highest values, respectively. The blend having 80.00%, 0.00%, and 20.00% IQBF, WF, and EFM were not significantly different (*p* > .05) in appearance from experimental runs 4 and 10 (without EFM), 14 and 16, respectively.

Sensory texture perception is a dynamic process influenced by senses of touch, sight but most importantly, by the oral processing of food in the mouth. The interrelationships between food structure, structural breakdown during oral processing, and sensory perception of texture are important for understanding the effects on satiation and satiety (Campbell et al., 2016) and also to food process development. The ratings of the texture of the blends (Table 5) ranged from 5.56 to 6.88, with experimental runs number 11 and 3 having the lowest and highest values, respectively. The blends having 60.00%, 0.00%, and 40.00% IQBF, WF, and EFM were not significantly different (*p* > .05) in texture from experimental runs 3, 4, 7, 8 (without WF), 10 (without EFM but with 40% WF), and 14 (with 20.4% WF), respectively. These implied that the texture of the biscuits might not have been adversely altered as a result of the replacement of the WF with either IQBF or EFM at these substitution levels. However, experimental runs 11 and 12, having only 100% IQBF had the lowest texture ratings. Values for taste ranged from 5.36 to 6.72. Experimental runs number 11 and 12 had the lowest taste ratings while experimental runs 14 and 15 had the highest values. The values for color ranged from 5.56 to 6.88 with the blends without EFM having relatively higher ratings, and notable exceptions were experimental runs 3 having up to 40% EFM. Overall acceptability: 5.48 to 6.88 with blends having 80.0%, 20.0%, and 0.00 IQBF, WF, and EFM adjudged to be most acceptable and is significantly different (*p* < .05) from most other blends but was not significantly different from blends having between 20.0% and 40.0%

### 3.2 Model description

This section deals with the discussion on an appropriate model that best describes the relationship between the response and the factor variables that were selected. Model fitness test that was conducted to identify the model that can best be used as a response predictor. The desire was for the selected model to have insignificant lack of fit in order to demonstrate that the model fits the data (Myers & Montogomery, 2002). The tested models were assessed for their adequacy for the experimental conditions, and the significant terms in each of the models were identified.

#### 3.2.1 Model fitness for each of the quality attributes

In selecting the appropriate model that best describes the relationship between the responses (nutritional, color, and texture properties) and the factor variables, it was assumed that a second‐order relationship would be relatively appropriate and that true function may be approximated by parabolic surface. This assumption was necessary because the actual form of a functional relationship between the response and the factor variables are unknown at this stage. If linear models are used for screening designs or robustness tests, each factor in the model only appears as a linear term. In this case, a linear term means a combination of a coefficient βi and a factor Xi. The interaction has similar uses as the linear but is more complex because of the additional interaction terms. An interaction term is the combination of two factors Xi and Xj with a conjoint coefficients ¯βij. Quadratic models are the most complex of the three basic model types and are used for the optimization processes. The quadratic model for the three factors model is as presented in equation (1) (Johnson & Nachtsheim, 1983). The fitness of each of the model was tested using the sequential model sum of squares (SMSS). The SMSS indicated the contributions of the linear, two‐factor interaction (2FI), quadratic and cubic polynomials terms to the totality of the model.

#### 3.2.2 Model testing

A quadratic model has suggested all the variables (not indicated on any of the Tables because of space constraint) except crude fat, estimation of total energy, calcium, and adhesiveness where the linear model was also suggested. These suggested models had the highest adjusted *R* ^2^ except where the model was aliased, cubic, or where there was no suggested model choice.

**Figure 2:**
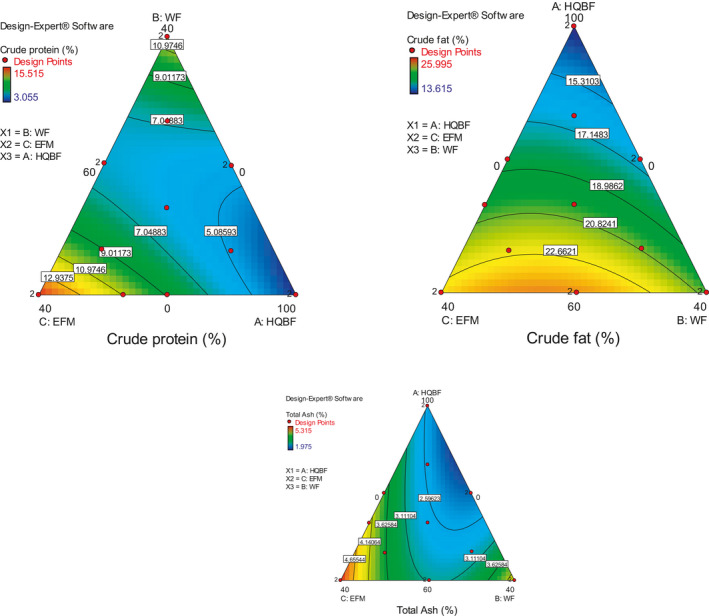
Contour plots of change in protein, fat, and ash contents of biscuit at different experimental conditions

**Figure 3:**
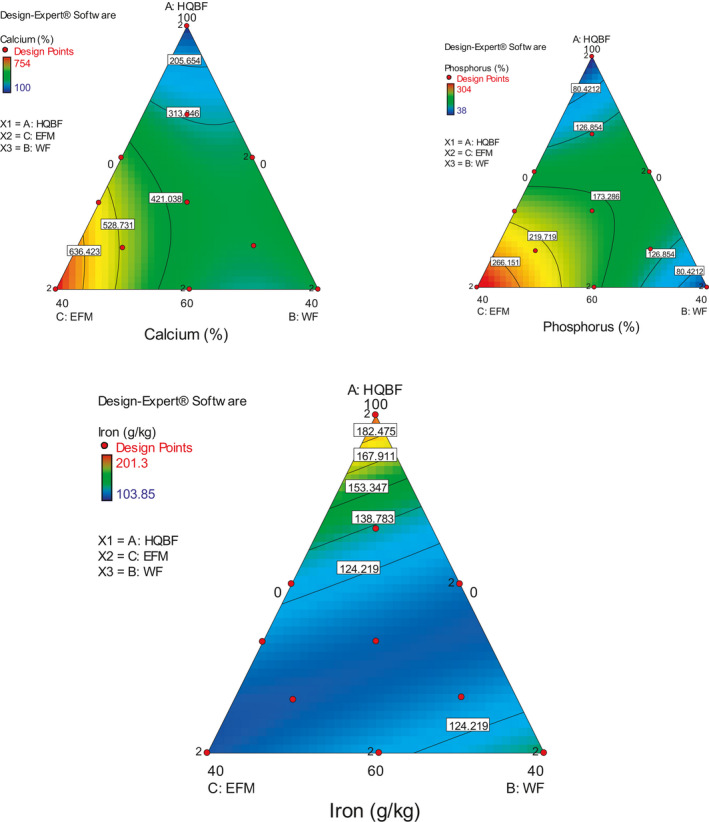
Contour plots of change in calcium, phosphorus, and iron of biscuit at different experimental conditions

**Figure 4:**
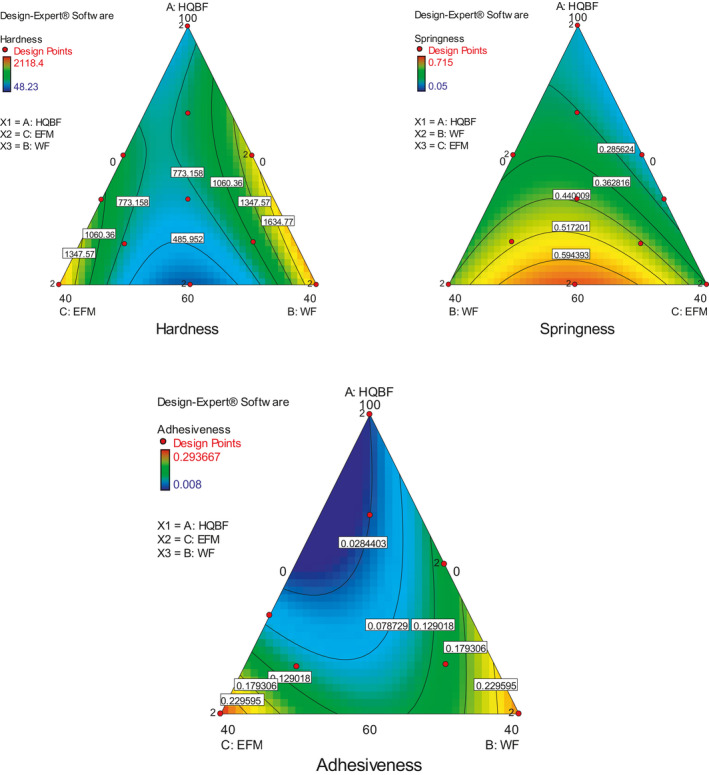
Contour plots of change in hardness, springiness, and adhesiveness of biscuit at different experimental conditions

The residual errors in each of the selected model were compared with the "Pure Error" from replicated design points to ascertain the extent of their lack of fitness. An insignificant lack of fit is indicated by a low probability value ("Prob > F"), low standard deviation, high adjusted *R*‐squared values, and a low predicted residual sum of squares (PRESS). These explained why these and other statistical parameters used in evaluating and selecting the best‐fitted model (Zen et al., 2015; Bakare et al., 2019). From the equation constructed for the best‐fitted model, positive coefficient presents a positive contribution toward the response and vice versa. Also, a contour plot and three‐dimensional response surface graphs (Figures 2, 3, 4) for each response were generated for a better explanation.

#### 3.2.3 Adequacy of the models for the experimental conditions

The models were assessed for their adequacy for the experimental conditions and the significant terms in each of the models were identified (Tables 6, 7, 8). These showed the relationships between each of the measured parameters (responses) of each of the quality attributes and the composite ingredients. It is important to reiterate that for most cases, we choose to test the significance or otherwise of the models based on quadratic relationships. This was to avoid the error of selecting cubic models with false assumption of significance and also to avoid under exploring the significance of a selected linear model. For instance, for the proximate and mineral properties (Table 6), the significance or otherwise of the models was tested based on quadratic relationships. The p‐value (Prob > F) and model *F*‐value for crude protein were 0.0101 and 5.62, respectively. The model was significant, and the significant terms in the model were A (IQBF), B (WF), C (EFM), and BC (WF * EFM). This indicated that three independent variables contributed significantly to the protein content of the biscuit. Moisture, which is also important to the storage stability of the biscuit, was influenced by the interactions between IQBF and WF as well as WF and EFM. In this study, particular attention was placed on the protein, fat, and ash contents of the biscuits as some of the core intents of the product development efforts. The contour plots (Figure 2) and the model equations can be explored to achieve the desired level of each of these macronutrients. Desired crude protein content can be extrapolated from the equation from equation (1).

(1) $$\begin{array}{c} \text{Crude protein (\textbackslash{}\% )}=3.12*A+12.00*B+14.90*C\\ ‐7.32*A*B‐0.50*A*C‐27.38*B*C \end{array}$$

where A, B, and C are as defined in the Tables.

**Table 6:** Baking and sensory properties of biscuits

ER	Spread ratio	Response variables				
		Appearance	Texture	Taste	Color	Overall acceptability
1	10.75 ± 0.60^bc^	6.84 ± 0.69^a^	6.64 ± 0.99^b^	6.68 ± 0.99^b^	6.64 ± 1.04^b^	6.88 ± 0.83^c^
2	11.21 ± 0.06^c^	6.60 ± 1.29^a^	6.12 ± 1.81^ab^	5.96 ± 1.72^ab^	6.44 ± 1.53^b^	6.04±`1.54 ^ab^
3	11.29 ± 0.06^c^	6.36 ± 1.85^a^	6.88 ± 1.13^b^	6.16 ± 1.77^ab^	6.52 ± 1.6^b^	6.56 ± 1.04^bc^
4	9.44 ± 0.88^abc^	6.96 ± 1.24^ab^	6.40 ± 1.19^b^	6.24 ± 1.51^ab^	6.88 ± 1.27^b^	6.60 ± 1.19^bc^
5	9.89 ± 0.72^abc^	6.72 ± 0.79^a^	6.60 ± 0.65^b^	6.60 ± 0.96^b^	6.64 ± 1.04^b^	6.52 ± 0.65^bc^
6	9.57 ± 0.00^abc^	6.84 ± 1.11^a^	6.12 ± 1.74^ab^	5.28 ± 1.62^a^	6.08 ± 1.35^ab^	6.04 ± 1.40^ab^
7	11.25 ± 0.32^c^	6.36 ± 1.85^a^	6.88 ± 1.13^b^	6.16 ± 1.77^ab^	6.52 ± 1.61^b^	6.56 ± 1.04^bc^
8	9.43 ± 0.60^abc^	7.12 ± 0.73^ab^	6.48 ± 1.12^b^	6.24 ± 1.70^ab^	6.92 ± 0.91^b^	6.80 ± 1.08^bc^
9	10.24 ± 0.54^bc^	6.84 ± 0.69^a^	6.64 ± 0.99^b^	6.68 ± 0.99^b^	6.64 ± 1.04^b^	6.88 ± 0.83^c^
10	9.44 ± 0.88^abc^	6.96 ± 1.24^ab^	6.40 ± 1.19^b^	6.24 ± 1.51^ab^	6.88 ± 1.27^b^	6.60 ± 1.19^bc^
11	9.63 ± 0.32^abc^	6.56 ± 1.33^a^	5.56 ± 2.02^a^	5.36 ± 1.98^a^	5.56 ± 1.56^a^	5.48 ± 1.64^a^
12	9.85 ± 0.00^bc^	6.56 ± 1.33^a^	5.56 ± 2.02^a^	5.36 ± 1.98^a^	5.56 ± 1.56^a^	5.48 ± 1.64 ^a^
13	10.75 ± 0.60^c^	6.44 ± 1.64^a^	6.28 ± 1.40^ab^	6.52 ± 1.23^b^	6.40 ± 1.50^b^	6.48 ± 1.58^bc^
14	9.78 ± 1.97^abc^	7.00 ± 0.71^ab^	6.80 ± 0.65^b^	6.72 ± 0.98^b^	6.68 ± 1.07^b^	6.76 ± 0.60^bc^
15	9.57 ± 0.00^abc^	6.80 ± 0.58^a^	6.32 ± 1.11^ab^	6.40 ± 1.47^b^	6.60 ± 1.19^a^	6.72 ± 0.68^bc^
16	9.78 ± 1.97^abc^	7.00 ± 0.71^ab^	6.80 ± 0.65^b^	6.72 ± 0.98^b^	6.68 ± 1.07^b^	6.76 ± 0.60^bc^

Note

Mean values with different superscripts within the column are significantly different at *p* < 0–05.

**Table 7:** Model properties and regression coefficients for proximate and mineral composition of biscuit

Parameters	Moisture content (%)	Crude protein (%)	Crude fat (%)		Crude fiber (%)		Total ash (%)	Total CHO (%)	Total energy (Kcal/kg)	Calcium (mg/100 g)	Magnesium (mg/100 g)	Potassium (mg/100 g)	Phosphorus (mg/100 g)	Sodium (mg/100 g)	Iron (mg/100 g)
Model selected *p*‐value* (Prob > F)	.0081#	.0101#	.0002#		.0008#		.0266#	.0009#	<.0001#	.0006#	<.0001#	.0115#	<.0001#	<.0001#	.0002#
Model *F*‐values	5.99	5.62	15.35		11.23		4.15	10.69	16.83	13.86	59.84	5.41	22.44	62.59	14.75
Remarks on models	*	*	*		*		*	*	*	*	*	*	*	*	*
Significant terms in the models	AB and BC	A, B, C, and BC	A, B, and C		A, B, C, and BC		A, B, C, and BC	A, B, and C	A, B, C, and AC	A, B, and C		AB	A, B, C, AB, and ABC	AB, AC, BC,ABC	A, B, C, AB, and AC
		Regression coefficients													
A – IQBF	3.86	3.12		13.47		1.37	2.53	75.63	4,575.3	167.77	58.92	572.18	32.20	330.32	197.04
B – WF	3.41	12.00		20.37		0.49	4.37	59.34	5,036.19	354.14	66.92	509.06	40.99	346.44	140.60
C – EFM	3.72	14.90		23.17		0.35	5.17	52.71	5,207.47	687.14	72.42	460.00	307.75	311.48	109.96
AB ‐ IQBF * WF	5.86	7.32		−0.87		1.18	−4.77	5.96	−71.68		−24.35	−523.86	590.74	−90.73	−218.42
AC – IQBF * EFM	3.32	−0.50		0.91		0.062	−0.28	−3.40	−674.16		−30.09	−172.21	37.14	83.76	−98.05
BC ‐ WF * EFM	6.73	−27.38		10.15		1.56	−6.70	15.72	−121.76		−34.83	−54.54	124.19	−65.57	−19.00
Mean	4.51	8.23		19.66		0.88	3.42	63.31	4,901.5	408.25	62.06	472.50	157.34	330.45	130.41
Std. Deviation	0.51	2.75		1.54		0.18	0.85	3.67	86.75	118.82	0.90	35.64	30.00	4.29	12.65
C.V. (%)	11.34	33.37		7.84		20.35	24.74	5.80	1.77	29.11	1.44	7.54	19.07	1.30	9.70
*R*‐Squared	0.7498	0.7376		0.8848		0.8489	0.6747	0.8424	0.8938	0.6807	0.9890	0.7299	0.9373	0.9895	0.8806
Adj *R*‐Squared	0.6247	0.6064		0.8271		0.7733	0.5120	0.7635	0.8406	0.6316	0.9725	0.5949	0.8956	0.9737	0.8209
Pred *R*‐Squared	0.5298	0.4353		0.7704		0.6806	0.2870	0.7155	0.8153	0.5605	−8.8811	0.5282	0.8098	−8.4519	0.8071
Adeq Precision	6.450	6.999		11.445		9.315	5.621	10.191	11.901	10.094	23.241	7.447	13.886	28.406	11.240
PRESS	4.92	162.52		47.36		0.68	15.68	243.52	1.31E+05	2.526E+005	4,322.37	22,190.77	24,593.33	99,122.31	2,586.39

**Not significant (*p* > .05 is not significant).

* Significant (*p* < .05 is significant).

^#^ Quadratic.

**Table 8:** Model properties and regression coefficients for color, textural properties, and spread ratio of biscuit

Parameters	*L* *	*a* *	*b* *	Hardness	Chewiness	Gumminess	Cohesiveness	Springiness	Adhesiveness	Deformation at Peak	Energy to Break	Spread ratio
		Model properties										
Model Selected *p*‐value* (Prob > F)	.0422#	.1096#	.2847#	.1004#	.7496#	.3646#	.7613#	.1941#	Linear .0319	Cubic .0044	.1650#	.9845
**Model *F*‐values**	3.54	2.42	1.46	**2.52**	0.53	1.23	0.51	1.83	5.17	13.59	2.00	0.12
**Remarks on models**	*	**	**	**	**	**	**		*	*	**	**
**Significant terms in the models**	BC	BC	None	BC	None	None	None	A, B, and C	A, B, and C	None		None
		Regression coefficients										
A—IQBF	46.06	5.7	14.82	615.4	69.23	380.23	0.67	0.24	1.011E−003	1.74	0.31	9.67
B—WF	36.85	9.45	15.49	1,921.98	472.51	934.24	0.44	0.44	0.25	1.70	1.43	9.64
C—EFM	47.25	6.14	17.71	1,704.92	387.21	993.76	0.62	0.37	0.22	1.73	1.08	9.78
AB—IQBF * WF	26.97	−8.5	11.53	1,180.82	680.02	1,484.22	0.11	3.66E−03	‐‐‐‐‐‐‐	−0.16	0.86	2.31
AC—IQBF * EFM	3.27	1.83	7.21	−1,093.04	−21.47	−760.64	−0.93	−0.32	‐‐‐‐‐‐‐	0.059	−0.68	0.29
BC—WF * EFM	34.44	−14.14	−6.82	−6453.25	−1,083.28	−3195.43	0.10	1.06	‐‐‐‐‐‐‐	−0.38	−4.24	1.45
Mean	46.86	5.98	16.66	1,085.26	290.48	644.2	0.54	0.39	0.15	1.70	0.73	9.92
Std. deviation	3.87	1.67	1.95	629.62	323.15	475.21	0.20	0.18	0.090	0.098	0.51	1.06
C.V. (%)	8.25	27.98	11.73	58.02	111.25	73.77	37.16	45.56	59.94	5.77	69.76	10.74
*R*‐squared	0.6389	0.5477	0.4221	0.5573	0.2094	0.3804	0.2041	0.4781	0.5348	0.2151	0.4994	0.0572
Adj *R*‐squared	0.4584	0.3216	0.1332	0.3359	−0.1859	0.0706	−0.1939	0.2171	0.4314	−0.1773	0.2491	−0.4143
Pred *R*‐squared	0.0214	−0.2142	−0.6923	0.2303	−0.2945	−0.0677	−0.5049	0.1089	0.2888	−0.3286	0.1737	−1.7284
Adeq Precision	5.796	5.031	2.955	4.458	2.038	2.967	2.107	4.115	5.557	2.165	3.928	0.91
PRESS	405.24	75.14	111.85	6.89E+06	1.710E+006	3.89E+06	0.75	0.54	0.11	0.16	4.32	32.81

* Significant (*p* < .05 is significant).

** Not significant (*p* > .05 is not significant).

^#^ Quadratic.

In contrast, most of the textural properties were tested on quadratic models exception being adhesiveness that was tested on linear. The model for hardness was not significant with p‐value and model *F*‐value of 0.1004 and 2.52 but had BC (WF * EFM) as a significant model term. Adhesiveness the only significant textural model properties, being linear had no interaction terms. Interestingly, models for chewiness, gumminess, and cohesiveness were not significant, had no significant terms and as such cannot be reckoned with as important texture attributes of this type of biscuit. These model descriptions implied that hardness, springiness, and adhesiveness were the only textural characteristics of this type of biscuit (Figure 4). Spread ratio (Table 7) was also not a significant model under texture quality, and this suggested that the shape of the biscuit may need to modify to enable better appreciation of the appearance and measurement of the spread ratio.

Except for appearance, the models for sensory properties (Table 8) were all significant, and the quadratic equation adequately explained all the parameters. Prominent interaction terms in all these variables were AB (IQBF * WF) in addition to the linear components of these models except for color where AC (IQBF * EFM) also featured. The model for sensory texture, in particular, was significant (*p* < .0013), and its relationship with adhesiveness (*n* = 16, *r* = 0.715 *p* = 0 0.009) was further reinforced by the fact that all the three components (IQBF, WF, EFM) and interactive effects of IQBF and WF were the significant terms in the model.

#### 3.2.4 Optimization of the Quality Attributes of the Biscuit

The goal of the optimization process in food product development is to determine the level of each variable from which a robust product with the desired quality characteristics may be obtained. In this study, nutritional improvement is a core objective without necessarily having to sacrifice the preferred quality attributes of biscuit. Specific attention was on protein, fat, and ash for the macronutrients while textural and sensory attributes of biscuits are key indices of acceptance by consumers.

**Figure 5:**
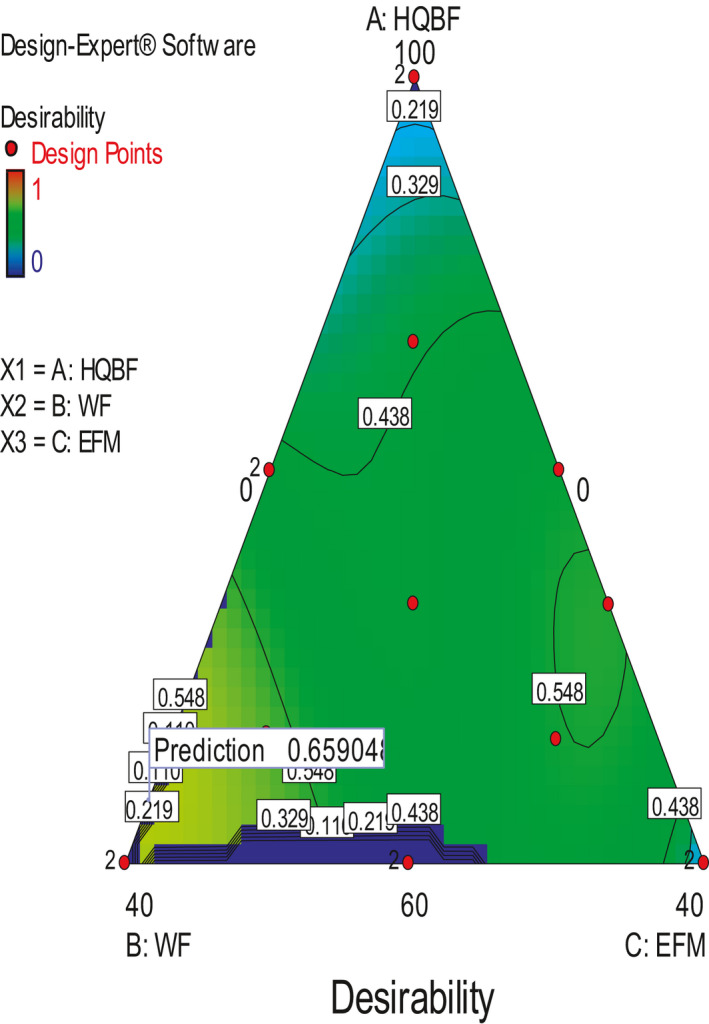
Desirability plot that indicated the region with the optimum combination of flour blends that falls within the constraints that were placed on the responses

The aim of the optimization experiment in this study was to determine the level of each of the flour blends that collectively would result in biscuit with not less than 10% protein contribution from the animal source, reduced fat content preferably not more than 15% and possibly maximum ash content. This aim was accomplished by placing constraints on the responses (Table 1). A desirability plot was generated by the software (Figure 5). It indicated the region with the optimum combination of flour blends that fall within the constraints that were placed on the responses. Five solutions were predicted, and the solution with the highest desirability index of 60.3% was selected for verification experiment. The predicted combinations (IQBF:WF:EFM) were as follows: 61.33:38.59:0.07, 60:39.51:0.49, 63.22:36.71:0.07, 66.15:33.84:0, 79.44:0.0:20.55 and desirability indices of 0.603, 0.598, 0.598, 0.576, 0.482, respectively. The verified quality attributes are presented in Table 9. Verification experiment conducted on the selected optimization solution showed that there was no significant difference (*p* > .05) between the predicted and verified values for the quality attributes (degree of freedom = 44, *t* value = 0.08 and *p*‐value 2‐tailed = 0.994) (Table 10).

**Table 9:** Model properties and regression coefficients for sensory attributes of composite biscuit

Parameters	Appearance	Texture	Taste	Color	Overall acceptability
Model properties					
Model Selected *p*‐value* (Prob > F)	.1211#	.0013#	.0050#	.0008#	.0045#
**Model *F*‐values**	2.32	9.79	6.86	11.12	**7.08**
**Remarks on models**	**	*	*	*	*
**Significant terms in the models**	None	A, B, C, and AB	A, B, C, and AB	A, B, C, and AC	A, B, C, and AB
Regression coefficients					
A—IQBF	6.61	5.60	5.28	5.57	5.50
B—WF	6.91	6.38	6.27	6.86	6.58
C—EFM	6.37	6.83	6.19	6.52	6.56
AB—IQBF * WF	−0.18	2.28	2.93	1.21	2.76
AC—IQBF * EFM	1.52	−0.34	0.56	2.10	1.01
BC—WF * EFM	0.91	0.29	1.77	−0.42	0.38
Mean	6.0.75	6.41	6.21	6.47	6.45
Std. deviation	0.20	0.21	0.29	0.20	0.26
C.V. (%)	2.98	3.24	4.61	3.04	4.02
*R*‐squared	0.5366	0.8303	0.7743	0.8475	0.7796
Adj *R*‐squared	0.3049	0.7455	0.6615	0.7713	0.6695
Pred *R*‐squared	0.0284	0.6071	0.5634	0.6345	0.4223
Adeq precision	4.387	9.747	7.942	10.665	8.062
PRESS	0.85	0.99	1.58	0.9315	1.76

* Significant (*p* < .05 is significant).

** Not significant (*p* > .05 is not significant).

^#^ Quadratic.

**Table 10:** Quality attributes of biscuit at optimization point

Quality characteristics	Predicted values	Verified values
Moisture (%)	4.43	4.49
Crude protein	11.52	10.41
Crude fat	20.65	17.59
Crude fiber	0.65	0.68
Total ash	4.38	2.05
Total CHO	58.41	64.41
Total energy (Kcal/Kg)	4,895.79	
Calcium mg/100 g	542.40	500.00
Magnesium mg/100 g	65.03	95.85
Potassium mg/100 g	456.64	462.64
Phosphorus mg/100 g	238.42	230.42
Sodium mg/100 g	356.62	357.62
Iron mg/100 g	114.52	120.52
Zinc mg/100 g		
Adhesiveness (N.s)	0.16	0.14
Chewiness (N)	294.28	295.28
Cohesiveness	0.44	0.45
Gumminess (N)	669.89	579.89
Hardness (N)	1,181.58	1,261.58
Springiness	0.26	0.24
Stringiness	1.27	1.27
L*	47.57	47.57
a*	6.38	6.38
b*	18.36	18.36
Overall acceptability	6.47	6.43

## 4 CONCLUSION

Biscuits of acceptable sensory quality were obtained from the sixteen blends of flour. Significant relationships were established between sensory texture and adhesiveness as well as between adhesiveness and the IQBF used for making the biscuits. The model for adhesiveness was the only texture parameter that correlated significantly with any of the composite ingredients (IQBF) and also with sensory texture an indication that specific functional properties might have a significant influence on the adhesive textural property of the biscuit made from the blends. The optimal combination of composite ingredients required to produce biscuits with desired quality attributes were 61.33% IQBF, 38.60% WF, and 0.07 EFM, respectively. The quality attributes include protein, fat, ash, iron, and calcium contents of 10.41%, 17.59%, 2.05%, 120.52 mg/100 g, and 500.00 mg/100 g, respectively.

## CONFLICT OF INTEREST

The authors declare that they do not have any conflict of interest.

## ETHICAL APPROVAL

Ethics approval was not required for this research.

## Data Availability

The data that support the findings of this study are available from the corresponding author upon reasonable request.
